# Fludarabine Inhibits K_V_1.3 Currents in Human B Lymphocytes

**DOI:** 10.3389/fphar.2017.00177

**Published:** 2017-03-31

**Authors:** Alicia de la Cruz, Alba Vera-Zambrano, Diego A. Peraza, Carmen Valenzuela, Juan M. Zapata, Gema Perez-Chacon, Teresa Gonzalez

**Affiliations:** ^1^Instituto de Investigaciones Biomédicas “Alberto Sols”, Consejo Superior de Investigaciones Científicas – Universidad Autónoma de MadridMadrid, Spain; ^2^Departamento de Bioquímica, Universidad Autónoma de MadridMadrid, Spain; ^3^Instituto de Investigación Hospital Universitario La Paz (IdiPaz)Madrid, Spain

**Keywords:** F-ara-A, fludarabine, K_V_1.3, chronic lymphocytic leukemia, B lymphocyte

## Abstract

Fludarabine (F-ara-A) is a purine analog commonly used in the treatment of indolent B cell malignancies that interferes with different aspects of DNA and RNA synthesis. K_V_1.3 K^+^ channels are membrane proteins involved in the maintenance of K^+^ homeostasis and the resting potential of the cell, thus controlling signaling events, proliferation and apoptosis in lymphocytes. Here we show that F-ara-A inhibits K_V_ currents in human B lymphocytes. Our data indicate that K_V_1.3 is expressed in both BL2 and Dana B cell lines, although total K_V_1.3 levels were higher in BL2 than in Dana cells. However, K_V_ currents in the plasma membrane were similar in both cell lines and were abrogated by the specific K_V_1.3 channel inhibitor PAP-1, indicating that K_V_1.3 accounts for most of the K_V_ currents in these cell lines. F-ara-A, at a concentration (3.5 μM) similar to that achieved in the plasma of fludarabine phosphate-treated patients (3 μM), inhibited K_V_1.3 currents by 61 ± 6.3% and 52.3 ± 6.3% in BL2 and Dana B cells, respectively. The inhibitory effect of F-ara-A was concentration-dependent and showed an *IC_50_* value of 0.36 ± 0.04 μM and a *n_H_* value of 1.07 ± 0.15 in BL2 cells and 0.34 ± 0.13 μM (*IC_50_*) and 0.77 ± 0.11 (*n_H_*) in Dana cells. F-ara-A inhibition of plasma membrane K_V_1.3 was observed irrespective of its cytotoxic effect on the cells, BL2 cells being sensitive and Dana cells resistant to F-ara-A cytotoxicity. Interestingly, PAP-1, at concentrations as high as 10 μM, did not affect the viability of BL2 and Dana cells, indicating that blockage of K_V_1.3 in these cells is not toxic. Finally, F-ara-A had no effect on ectopically expressed K_V_1.3 channels, suggesting an indirect mechanism of current inhibition. In summary, our results describe the inhibitory effect of F-ara-A on the activity of K_V_1.3 channel. Although K_V_1.3 inhibition is not sufficient to induce cell death, further research is needed to determine whether it might still contribute to F-ara-A cytotoxicity in sensitive cells or be accountable for some of the clinical side effects of the drug.

## Introduction

F-ara-A (Fludarabine, 9-β-D-arabinofuranosyl-2-fluoroadenine) is the most extensively used purine analog in the treatment of indolent B cell malignancies. It is broadly used in the treatment of chronic lymphocytic leukemia (CLL) either alone or in combination therapy, although its use has been extended to other B lymphoproliferative disorders, including follicular lymphoma and mantle cell lymphoma (Lenz et al., 2004; Ungerechts et al., 2007). F-ara-A is also used in conditioning regimens before stem-cell transplantation therapies (Anderlini et al., 2016; Laport et al., 2016). F-ara-A is administered to patients as its monophosphorylated form (fludarabine phosphate), which is a non-membrane permeable prodrug that requires to be dephosphorylated to enter the cells, where it is phosphorylated to the active triphosphate form, 9-β-D-arabinofuranosyl-2-fluoroadenine-5′-triphosphate (F-ara-ATP) (Kano et al., 2000; Gandhi and Plunkett, 2002). It has been demonstrated that the cellular influx of the drug occurs preferentially in active leukemia cells, although it also enters into normal cells (Barrueco et al., 1987). F-ara-A seems to interfere with DNA synthesis, by inhibiting DNA polymerases, primases and the ribonucleotide reductase. However, in quiescent cells, such as CLL cells, its cytotoxic function has been associated to the inhibition of DNA transcription and RNA translation (Huang et al., 2000).

Recently, it has become evident the importance of ion channels in lymphocyte function and they are considered suitable therapeutic targets for treating leukemia and lymphoma. Ion channels play a pivotal role setting the resting membrane potential and, therefore, controlling Ca^2+^, Mg^2+^, and Zn^2+^ cell concentrations, thus regulating a plethora of cell functions (Feske et al., 2012). The voltage-dependent potassium channel K_V_1.3 is a membrane spanning protein complex with a hydrophilic pore that opens under depolarization of the cell generating a K^+^ efflux. K_V_1.3 has relevant functions in the control of cell proliferation, apoptosis, and cell volume regulation (Feske et al., 2012). In addition, there is evidence indicating that K_V_1.3 channels are more expressed in different types of leukemias and lymphomas, compared to that of normal B lymphocytes, with the levels of K_V_1.3 expression correlating with increased proliferation rates and overall aggressiveness of these tumors (Comes et al., 2013; Leanza et al., 2013).

In this report, we show that F-ara-A, one of the chemotherapeutic drugs most extensively used in the treatment of indolent B cell malignancies, significantly inhibits K_V_1.3 currents in human B lymphocytes.

## Materials and Methods

### Drugs

F-ara-A (Sigma–Aldrich) was dissolved in DMSO to yield stock solution of 35 mM from which further dilutions in the patch-clamp external solution were made. The K_V_1.3 selective inhibitor 5-(4-phenoxybutoxy)psoralen (PAP-1; Sigma–Aldrich) was dissolved in DMSO to yield stock solution of 1 mM from which further dilutions in the patch-clamp external solution were made.

### Cell Culture and Transient Transfection

The Burkitt’s lymphoma BL2 and the Epstein-Barr virus (EBV)-transformed lymphoblastoid Dana cell lines were cultured in RPMI medium supplemented with 10% FBS (Gibco), 2 mM L-glutamine, 100 U/ml of penicillin and 100 μg/ml streptomycin (Sigma–Aldrich). HEK-293 and COS-7 cells were cultured in DMEM medium supplemented with 10% FBS, 100 U/ml of penicillin and 100 μg/ml streptomycin. *Ltk^-^* cells were cultured in DMEM supplemented with 10% FBS, 0.25 mg/ml G418 (Gibco) and 0.05 mg/ml gentamicin. Cells were maintained at 37°C under a 5% CO_2_ atmosphere. HEK-293 and COS-7 cells were co-transfected with K_V_1.3-pEYFP and a reporter plasmid expressing CD8 using Fugene 6 (Promega Biotech Iberica) following the manufacturer’s directions. Stably transfected *Ltk^-^* cells with the gene encoding the expression of K_V_1.5 channel were a gift of Dr. M. M. Tamkum (Colorado State University, USA). Prior to electrophysiological experiments, transfected HEK-293 cells were incubated with polystyrene microbeads coated with an anti-CD8 antibody (Dynabeads CD8, Invitrogen), as previously described (Gonzalez et al., 2002; Macias et al., 2010).

### Quantitative PCR (Q-PCR)

Total RNA was extracted with TRI reagent solution and the PureLink^TM^ RNA mini kit and 1 μg of RNA was reverse transcribed using 2 U Superscript II reverse transcriptase (all from Thermo Fisher Scientific), following the manufacturer’s directions. Q-PCR was carried out by the Service of Genomics from our institute by means of the Applied Biosystems 7900 HT Fast Real-Time PCR System using a SYBR Green probe. Primers used for K_V_1.3 were: 5′ctggttctccttcgaactgc3′ and 3′gagaaggtggctttgctagg5′. The relative RNA expression was calculated in relation to 18S by the application of the Pfaffl analysis method (Pfaffl, 2001).

### Immunoblot Analysis

Murine and human cell line cells were lysed in modified Laemmli buffer (125 mM Tris pH 6.8, 4% SDS, and 20% glycerol) supplemented with a mixture of protease and phosphatase inhibitors. Lysates were sonicated and protein concentration was determined by the bicinchoninic acid method (Pierce). Protein samples (50 μg per condition) were supplemented with 2.5% 2-mercaptoethanol and 0.004% bromophenol blue, and subjected to SDS-PAGE analysis and immunoblotting. The antibodies used were: anti-K_V_1.3 APC-002 (1:200), anti-K_V_1.5 APC-004 (1:500) (Alomone), anti-ERK2 (Santa Cruz Biotech), and horseradish peroxidase-conjugated anti-rabbit (BioRad Laboratories). Proteins were detected by chemiluminescence and exposure on film. ERK2 expression was used as an internal loading control.

### Analysis of Cell Viability

Cells (10^6^ cells ml^-1^; 100 μl per well) were incubated in 96-well microtiter plates and cultured in the presence of the indicated concentrations of F-ara-A. After 48 h of culture, cell viability was assessed by using the kit CellTiter 96© AQUEOUS Assay, following the manufacturer’s instructions. The spectrophotometric absorbance of each sample was measured at 490 nm using the BioTek Synergy Mx microplate reader (BioTek Instruments).

### Electrophysiological Recordings and Data Acquisition

The extracellular solution contained the following (in mM): NaCl 145, KCl 4, CaCl_2_ 1.8, MgCl_2_ 1, HEPES-Na 10, and glucose 10 (adjusted to pH 7.40 with NaOH). For recording on B lymphocytes, the intracellular pipette filling solution contained the following (in mM): KF 140, MgCl_2_ 2, CaCl_2_ 1, HEPES-K 10 and EGTA-K 11 (pH 7.2 with KOH). For HEK-293 cells, the intracellular solution contained (in mM): aspartate-K 80, KCl 42, phosphocreatine 3, KH_2_PO_4_ 10, ATP-Mg 3, HEPES-K 5 and EGTA-K 5 (pH 7.25 with KOH). Currents were recorded using the whole-cell configuration of the patch-clamp technique with a patch-clamp amplifier (Axopatch-200B, Molecular Devices) and were stored on a personal computer with a Digidata 1440A analog-to-digital converter (Molecular Devices). PClamp 10 software (Molecular Devices) was used for both data acquisition and analyses. Currents were recorded at room temperature (21–23°C) at a stimulation frequency of 0.1 Hz and were sampled at 4 kHz after anti-alias filtering at 2 kHz. The average pipette resistance ranged from 3 to 4 MΩ. Gigaohm seal formation was achieved by suction (2–5 GΩ). After seal formation, cells were lifted from the bottom of the bath, and the membrane patch was ruptured with a brief additional suction. Origin 8.5 (OriginLab Co) and the Clampfit utility of pClamp10 were used to perform least squares fitting and data presentation. Degree of inhibition obtained for each drug concentration [D] was used to calculate the *IC_50_* and *n_H_* by fitting to a Hill equation:

$$y=\frac{1}{1+\frac{IC_{50}}{{\left[D\right]}^{nH}}}$$

The current-voltage relationships were obtained by the application of 250-ms pulses from -80 to +40 mV, in 10 mV increments, from a holding potential of -80 mV followed by pulses to -40 mV to record deactivating tail currents, every 45 s to allow total recovery from inactivation of the channels. Inactivation was fitted to a monoexponential process:

$$y=Ae^{\left(\frac{-t}{\tau }\right)+C}$$

Where *τ* is the system time constant, *A* is the amplitude and *C* is the baseline value. Frequency-dependent decay of the current was analyzed applying a train of 10 pulses from -80 to +40 mV of 250 ms in duration at a frequency of 1 Hz. The peak current of each pulse was normalized to the peak current of the first pulse and plotted *versus* the pulse number.

### Statistical Analysis

GraphPad Prism v.6 was used for statistical analysis. The data are presented as mean ± SEM. Comparisons were performed by a Student *t*-test and statistical significance was set at *P* < 0.05. The data and statistical analysis comply with the recommendations on experimental design and analysis in pharmacology (Curtis et al., 2015).

## Results

### Expression of K_V_1.3 Ion Channel in BL2 and Dana Cells

**FIGURE 1:**
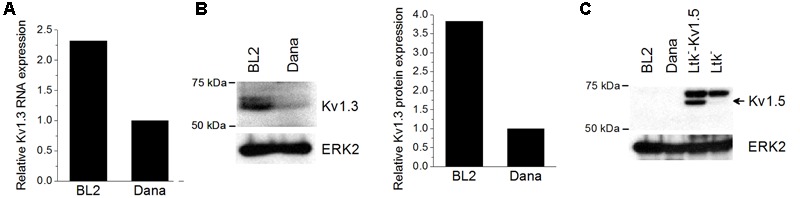
**K_V_1.3 and K_V_1.5 expression in BL2 and Dana cells.** RNA and protein were extracted from the different cell lines as described in Section “Materials and Methods.” **(A)** Basal K_V_1.3 gene expression was determined by Q-PCR using specific primers and the relative expression was calculated as the ratio of the target mRNA to 18S mRNA by the application of the Pfaffl analysis method. For protein analysis, a total of 50 μg of protein for each sample were subjected to 8% SDS-PAGE and immunoblotting with specific antibodies against K_V_1.3 **(B)** and K_V_1.5 **(C)**. ERK2 was used as a loading control. *Ltk^-^* cells with or without the plasmid expressing K_V_1.5 channels were used as a positive or negative control of K_V_1.5 expression, respectively.

Firstly, we analyzed the expression levels of K_V_1.3 mRNA in the Burkitt’s lymphoma BL2 cell line and in the Dana lymphoblastoid cell line by Q-PCR (**Figure 1A**). Both cell lines expressed K_V_1.3 mRNA, but BL2 exhibited 2-fold more mRNA than Dana cells. This expression pattern was also evident at protein level, K_V_1.3 being more prominently expressed in BL2 cells (**Figure 1B**). Recently, it has been also described the expression and functional relevance of another potassium channel, K_V_1.5, in B lymphocytes (Vallejo-Gracia et al., 2013). However, we did not observed K_V_1.5 protein expression in either BL2 or Dana cells (**Figure 1C**).

### Electrophysiological Characterization of K_V_ Currents Recorded on BL2 and Dana Cells

**FIGURE 2:**
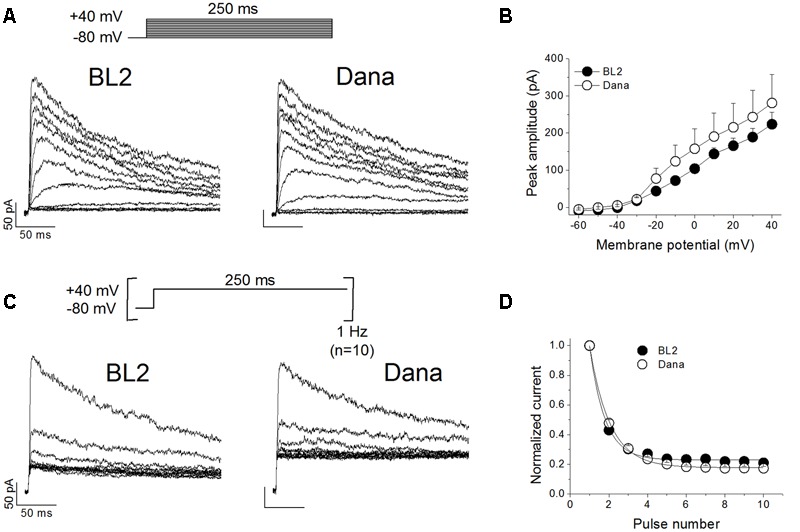
**Electrophysiological characteristics of the K_V_ current recorded on BL2 and Dana B lymphocytes. (A)** Original current traces obtained on BL2 and Dana cells after 250 ms depolarisations from –80 mV to +40 mV, in 10 mV steps, every 45 s. **(B)** Current–voltage relationships obtained after plotting the maximum amplitude of the current *versus* the membrane potential, for BL2 and Dana cells. **(C)** Use-dependent decay of the current. BL2 or Dana cells were depolarized from –80 to +40 mV during 250 ms at a frequency of 1 Hz. **(D)** Plot of the maximum peak current recorded with the protocol shown in **(B)** *versus* the number of pulse. Each point represents the mean of 5–17 experiments.

We analyzed the membrane passive properties of these cell lines. The resting membrane potential values were -10.2 ± 1.8 mV, *n* = 23, and -9.1 ± 1.7 mV, *n* = 35, *P* > 0.05, for BL2 and Dana cells, respectively. Cell capacitance values, which are proportional to the cell surface area, were also similar in both cells lines (6.9 ± 0.8 pF, *n* = 26 for BL2 cells, and 5.7 ± 0.4 pF, *n* = 31 for Dana cells, *P* > 0.05). We recorded and characterized the K_V_ currents elicited by the channels present in BL2 and Dana B cells. **Figure 2A** shows representative current traces recorded in each cell line after applying the voltage protocol shown on the top of the panel (see Materials and Methods). As expected for a K_V_1.3-driven current, the recorded currents exhibited a fast activation and a slow and incomplete inactivation. Moreover, these currents were abolished by the selective K_V_1.3 inhibitor PAP-1 (0.2 μM) (93.3 ± 1.2%, *n* = 6, for BL2 cells; 86.2 ± 1.4%, *n* = 5, for Dana cells), indicating that plasma membrane K_V_1.3 channels are responsible for most of the K_V_ current in these cell lines. K_V_ current was apparent at potentials positive to -40 mV (**Figure 2B**), reaching a similar peak current amplitude in both cell lines (317.7 ± 37.9 pA, *n* = 28, and 302.3 ± 29.2 pA, *n* = 31, *P* > 0.05, for BL2 and Dana cells, respectively). The inactivation degree was also similar (59.7 ± 2.8%, *n* = 28, and 59.8 ± 2.1%, *n* = 31, *P* > 0.05, for BL2 and Dana cells, respectively). However, the inactivation kinetics was slightly faster in BL2 cells (133.9 ± 7.0 ms, *n* = 27, *versus* 163.2 ± 8.8 ms, *n* = 30, *P* < 0.05, for BL2 and Dana cells, respectively). One of the main characteristics of K_V_1.3 channels is its slow recovery from inactivation that induces a use-dependent decay of the current when cells are stimulated at frequencies faster than 0.2 Hz. **Figure 2C** shows representative traces of current recorded on each cell line after applying a train of pulses at +40 mV at 1 Hz. In both cell lines, the peak current decreased in a monoexponential manner to a similar extent (80.9 ± 1.6%, *n* = 23, and 83.4 ± 1.2%, *n* = 20, *P* > 0.05, for BL2 and Dana cells, respectively) (**Figure 2D**). In summary, both cell lines exhibited similar currents with characteristic K_V_1.3 features.

### F-ara-A Inhibits the K_V_1.3 Current Recorded on BL2 and Dana Cells

**FIGURE 3:**
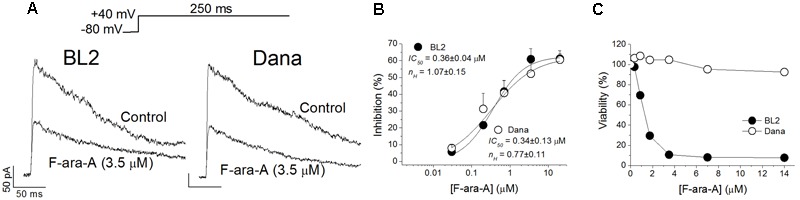
**Effects of F-ara-A on BL2 and Dana K_V_1.3 currents and cell viability. (A)** Superimposed current records obtained after depolarization of the cells to +40 mV from –80 mV during 250 ms, in the absence or in the presence of F-ara-A (3.5 μM) on BL2 or Dana cells. **(B)** Concentration-effect curves obtained after plotting the inhibition of the maximum current induced by F-ara-A *versus* the concentration of the drug and fitting these values to a Hill equation. The *IC_50_* and *n_H_* values are noted in the figure. Each point represents the mean of 5–10 experiments. **(C)** BL2 and Dana cells were cultured in triplicates in medium containing vehicle control (0.02% DMSO) or with the indicated F-ara-A concentrations (0.35–14 μM). After culturing for 48 h the percentage of viable cells was determined by a colorimetric assay considering the control condition as 100% viability.

**FIGURE 4:**
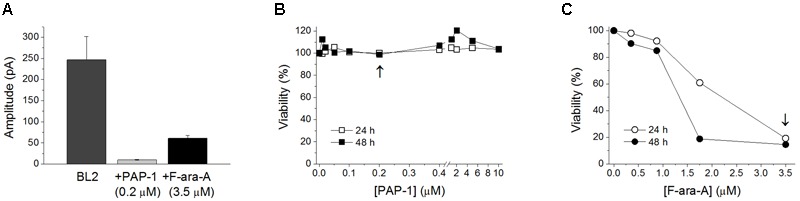
**Effects of PAP-1 and F-ara-A on BL2 current amplitude and cell viability. (A)** K_V_1.3 current amplitude in BL2 cells in control conditions and after perfusion with PAP-1 (0.2 μM) or F-ara-A (3.5 μM). Concentration-dependent effect of PAP-1 **(B)** or F-ara-A **(C)** on BL2 cell viability. After 24 and 48 h, the percentage of viable cells was assessed. Vehicle-treated cells provided the value for 100% viability. The arrows indicate the concentration of the drug tested in the electrophysiological experiments showed in **(A)**.

Cell perfusion with F-ara-A (3.5 μM) induced a decrease of the peak K_V_1.3 current by 61.0 ± 6.3% (*n* = 10) and 52.3 ± 6.3% (*n* = 6, *P* > 0.05) for BL2 and Dana cells, respectively (**Figure 3A**). Steady-state inhibition was achieved after 6.9 ± 2.0 min (BL2 cells) and 13.9 ± 2.0 min (Dana cells). This inhibitory effect was concentration-dependent, with similar *IC_50_* and *n_H_* values (**Figure 3B**). For BL2 cells, F-ara-A presented an *IC_50_* value of 0.36 ± 0.04 μM and an *n_H_* value of 1.07 ± 0.15, while in Dana cells, these values were 0.34 ± 0.13 μM and 0.77 ± 0.11. F-ara-A (3.5 μM) did not modify any other characteristic of the current such as the use-dependent decay at 1 Hz, the inactivation degree or the inactivation kinetics (**Table 1**). Remarkably, the inhibition of the K_V_1.3 currents by F-ara-A was similar in cells highly sensitive to F-ara-A (BL2) and in cells refractory to this drug (Dana) (**Figure 3C**), suggesting that K_V_1.3 current inhibition by F-ara-A is not sufficient to induce cell death. To further assess the effect of K_V_1.3 inhibition on cell viability, we analyzed the effect of the K_V_1.3 selective inhibitor PAP-1 (*IC_50_* for K_V_1.3 inhibition ∼2 nM) on BL2 cell viability (**Figure 4**), comparing it with that of F-ara-A. Our data show that 0.2 μM PAP-1 abrogated K_V_1.3 activity while 3.5 μM F-ara-A inhibited about 75% of K_V_1.3 activity (**Figure 4A**). However, PAP-1 concentrations up to 10 μM had no effect on cell viability (**Figure 4B**), while F-ara-A killed 80% of the cells (**Figure 4C**), thus suggesting that, at least in BL2 and Dana cells, K_V_1.3 inhibition is not cytotoxic.

**Table 1:** K_V_1.3 current characteristics on BL2 and Dana cells in control conditions and with F-ara-A (3.5 μM).

	BL2		Dana	
	Control	F-ara-A	Control	F-ara-A
Inactivation time constant (ms)	127.4 ± 9.4	107.0 ± 9.7 *(7)*	184.8 ± 26.6	130.5 ± 15.8 *(6)*
Degree of inactivation (%)	51.8 ± 3.7	62.6 ± 7.2 *(11)*	52.7 ± 6.2	67.5 ± 3.1 *(6)*
Use-dependent inactivation (%)	82.3 ± 3.0	72.8 ± 6.8 *(8)*	84.9 ± 2.7	87.9 ± 2.9 *(6)*

*Data is shown as mean ± SEM. Number in parentheses is the number of experiments. In all the cases, the comparison between control conditions and in the presence of the drug was not significant (*P* > 0.05).*

### Effects of F-ara-A on K_V_1.3 Channels Expressed in HEK-293 Cells

**FIGURE 5:**
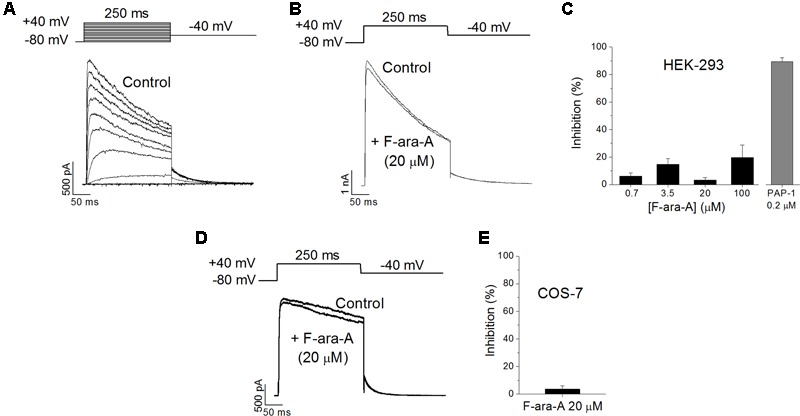
**Effects of F-ara-A on K_V_1.3 channels expressed on heterologous systems. (A)** Original current records of HEK-293 cells transfected with K_V_1.3 channels. Current was elicited after depolarizing the cell from –80 mV to +40 mV, in 10 mV steps, during 250 ms, every 45 s. Following each depolarization, cell was repolarised to –40 mV to record tail currents. **(B)** Currents recorded at +40 mV, from -80 mV, before and after perfusion with F-ara-A (20 μM). **(C)** Concentration dependence of K_V_1.3 current inhibition on HEK-293 cells induced by different concentrations of F-ara-A and by PAP-1 (0.2 μM). **(D)** Currents recorded at +40 mV, from –80 mV, from a COS-7 cell transfected with K_V_1.3 before and after perfusion with F-ara-A (20 μM). **(E)** Degree of inhibition on transfected COS-7 cells. *n* = 5–8.

To check if the inhibition of the K_V_1.3 function induced by F-ara-A observed in B cells is due to a direct effect of the drug on the channel, we transiently transfected HEK-293 cells with a plasmid encoding the human K_V_1.3 channel. We had chosen the HEK-293 cell line because it is a well-established model to study ion channel function; they exhibit an endogenous K_V_ current of very small amplitude (in the pA range *versus* the nA range of the transfected ones, **Figure 5**); and they do not express K_V_β subunits (Uebele et al., 1996). A representative family of traces recorded on an HEK-293 cell transfected with the K_V_1.3 cDNA is shown in **Figure 5A**. In these transfected cells, F-ara-A exhibited very low potency to inhibit this current, causing a 3.2 ± 1.9% reduction of K_V_1.3 current at 20 μM (*n* = 7) (**Figures 5B,C**) after 27.6 ± 3.5 min of perfusion with the drug. However, this current was abolished by the selective inhibitor PAP-1 at 0.2 μM (89.5 ± 2.8% inhibition, *n* = 5). This result indicates that F-ara-A had little effect on K_V_1.3 activity in HEK-293 cells ectopically expressing this channel compared to the inhibition achieved in endogenous K_V_1.3 channels in B lymphocytes. To assess whether this lower potency effect of F-ara-A was specific of the HEK-293 cells, we also tested F-ara-A on COS-7 cells transfected with K_V_1.3, obtaining similar results (**Figures 5D,E**). At 20 μM, F-ara-A had almost no inhibitory effect on the current (3.6 ± 2.3%, *n* = 5). The fact that PAP-1 but not F-ara-A inhibits K_V_1.3 activity in K_V_1.3 transfected cells suggests that the effect of F-ara-A on the channel is not due to a direct block of the channel protein.

## Discussion

In this report, we show that K_V_1.3 is the most prominent member of the voltage-gated K channels expressed in Burkitt’s lymphoma BL2 cells and on EBV-transformed lymphoblastoid Dana B cells. We have also characterized the K_V_ currents from BL2 and Dana cells. The K_V_ current recorded in these cell lines was abolished by the K_V_1.3 selective inhibitor PAP-1, confirming that it is carried by K_V_1.3 channels. Furthermore, the main features of the recorded current were the expected for a K_V_1.3-driven current and were similar in both cell lines: fast activation, slow inactivation and use-dependent inactivation. Our data indicate that BL2 cells express more K_V_1.3 mRNA and protein than Dana cells. However, the larger total K_V_1.3 protein expression on BL2 cells does not correlate with higher K_V_1.3 current amplitude compared to Dana cells, since the mean peak current amplitude was similar in both B cell lines despite the differences in protein expression. This result might indicate that the amount of functional K_V_1.3 channels in the plasma membrane of BL2 and Dana cells are similar, and that K_V_1.3 surplus in BL2 may be located at cytosolic reservoirs (i.e., mitochondria). However, there are other non-excluding possibilities, such as a different composition of the modulatory machinery in each cell line. In this regard, it is known that K_V_1.3 channels are modulated by different kinases (PKA, PKC and tyrosine kinases), which decrease their current amplitude without modifying other current characteristics (Payet and Dupuis, 1992), as well as by other proteins, such as the K_V_β subunits (McCormack et al., 1999). Therefore, BL2 and Dana cells may have different repertoire or different levels of expression of these modulatory proteins that may change current amplitude.

It is noteworthy that other Burkitt’s lymphoma cell lines, such as Raji and Ramos, also express K_V_1.5 channels (Vallejo-Gracia et al., 2013) and that K_V_1.3 and K_V_1.5 can form heterotetramers with particular current characteristics, as it have been described in macrophages (Vicente et al., 2003; Vicente et al., 2006; Villalonga et al., 2010; Moreno et al., 2013). However, our analyses assessing the presence of K_V_1.5 family member in BL2 and Dana cells failed to detect K_V_1.5 in any of them, thus ruling out that heterotetramers could explain the differences in the current between these two cell lines.

We also describe for the first time that F-ara-A is an inhibitor of K_V_1.3 channel activity. F-ara-A is the purine analog most extensively used in indolent B cell malignancies, such as CLL, follicular lymphoma and mantle cell lymphoma (Gandhi and Plunkett, 2002). F-ara-A inhibited the K_V_1.3 current in BL2 and Dana B cell lines with an *IC_50_* ∼0.3 μM. This concentration is 10 times lower than the concentration of F-ara-A (3 μM) achieved in patients plasma after being administered the standard therapeutic dose of fludarabine-phosphate (25–30 mg/m^2^/day) (Gandhi and Plunkett, 2002), thus indicating that inhibition of the K_V_1.3 current by F-ara-A is achievable at clinical dosages.

To determine if the observed inhibition was due to a direct effect of F-ara-A on the K_V_1.3 channel protein, we expressed K_V_1.3 channels on HEK-293 and COS-7 cells. The inhibitory effect of F-ara-A on ectopically expressed K_V_1.3 cells was very low, although PAP-1 could efficiently inhibit the current of ectopic K_V_1.3 channels, thus indicating that the mechanism of action of PAP-1 and F-ara-A on the K_V_1.3 channel is different. This result points to an indirect action of F-ara-A on the channel activity rather than to a direct block of the channel pore. A similar mechanism has been described for 15-epi-lipoxin-A_4_, which efficiently decreases the K_V_ current magnitude in macrophages but has no effect on K_V_1.3, K_V_1.5 and K_ir_2.1 when ectopically expressed in HEK-293 cells (Moreno et al., 2013). There are different possible explanations to the different sensitivity of K_V_1.3 channels to F-ara-A in B cells compared to that of ectopic systems. One could be that the channels expressed in heterologous systems adopt a conformation that hinders the binding of the drug. In addition, it would be possible that the rate of conversion of the prodrug to the drug catalyzed by the deoxycytidine kinase (Gandhi and Plunkett, 2002) is slower in HEK-293 and COS-7 cells than in B lymphocytes. Another possibility would be that the drug is acting on a protein or signaling pathway that is relevant for K_V_1.3 activity that is present in lymphocytes but not in HEK-293 or COS-7 cells. In this regard, K_V_ channels assemble with other membrane and cytosolic proteins in the plasma membrane forming regulatory and signaling complexes or channelosomes (David et al., 2012; Velez et al., 2016), and their localization in specific membrane microdomains (lipid rafts) is crucial for their function (Martens et al., 2004; Moreno et al., 2015). Indeed, it is known that K_V_1.3 channels target caveolar structures in the cell membrane that regulate their activity (Vicente et al., 2008; Perez-Verdaguer et al., 2016a). Thus, the K_V_1.3 channelosome in HEK-293 and COS-7 cells may lack a component that is present in B lymphocytes and that is required for the inhibition of the current induced by F-ara-A. Similar arguments have been proposed for the reduced sensitivity of K_V_1.3 channels to modulation by PKA and PKC in HEK-293 cells compared to T lymphocytes (Martel et al., 1998).

K_V_1.3 channels are currently considered as new targets for the treatment of cancer. K_V_1.3 expression is increased in some human malignancies, such as breast and colon cancer (Perez-Verdaguer et al., 2016b) and CLL (Leanza et al., 2013), and K_V_1.3 inhibition has been shown to induce cell apoptosis (Chandy et al., 2004; Wulff et al., 2009; Teisseyre et al., 2015) and cell proliferation blockage (Cahalan and Chandy, 2009). However, our results showing that F-ara-A is similarly effective inhibiting the K_V_1.3 currents generated by channels located in the plasma membrane of B cells that are either sensitive (BL2) or resistant (Dana) to F-ara-A, suggest that this activity is not sufficient to explain the cytotoxic activity of F-ara-A. Furthermore, we show that the inhibition of K_V_1.3 channels in BL2 cells with a selective K_V_1.3 inhibitor (PAP-1) at concentrations ranging from 10 nM to 10 μM does not induce cell death (*IC_50_* for K_V_1.3 inhibition ∼2 nM). This result indicates that K_V_1.3 channel inhibition is not cytotoxic in these cells. This result is in contrast with a previous study in which PAP-1 induced death in CLL cells (Leanza et al., 2013). However, the concentration of PAP-1 used in this study was higher (20 μM) and it was used in combination with inhibitors of the multidrug resistance pumps.

Nevertheless, we cannot rule out that K_V_1.3 inhibition by F-ara-A might contribute to the ability of the drug to promote cell death in F-ara-A-sensitive cells, such as BL2, or whether this inhibitory activity might be responsible for some of the clinical side effects of the drug. It is noteworthy that, despite the broad clinical usage of F-ara-A, its mechanism of action is not fully understood, although the interference with DNA and RNA synthesis would likely underlie its cytotoxic activity.

In summary, the results described herein unveil the effect of F-ara-A on K_V_1.3 channel activity in human B cell lymphoma and lymphoblastoid cells and open new venues to understand both the antineoplastic effect and the clinical side effects of the drug.

## Author Contributions

TG and GP-C designed research; AdC, AV-Z, DAP, and TG performed and analyzed electrophysiological experiments; GP-C designed, performed and analyzed molecular biology and viability experiments; GP-C, JMZ, CV, and TG interpreted and discussed the data; TG and JMZ wrote the paper.

## Conflict of Interest Statement

The authors declare that the research was conducted in the absence of any commercial or financial relationships that could be construed as a potential conflict of interest.
